# What affects people’s use of and attitudes toward gender-inclusive language? A literature review

**DOI:** 10.3389/fpsyg.2025.1657753

**Published:** 2025-09-08

**Authors:** Elena Lange, Lisa von Stockhausen

**Affiliations:** ^1^Department of Psychology, University of Bielefeld, Bielefeld, Germany; ^2^Department of Psychology, Institute of Psychology, University of Duisburg-Essen, Essen, Germany

**Keywords:** gender-inclusive language, gender-fair language, sexist language, attitudes, language use

## Abstract

This review synthesizes research on factors shaping attitudes toward and use of gender-inclusive language across linguistic, cultural, and methodological contexts. Sexist beliefs consistently predict negative attitudes and lower usage. Neither gender nor age per se are reliable predictors. Use of inclusive language has risen over time, driven by shifting norms and increased exposure. Political and social environments play a role, with progressive contexts and early equality legislation linked to greater support. Situational factors—such as conversational setting and perceived audience views—and political orientation influence language choices, with liberal individuals and institutions using inclusive forms more often. People with high and low education levels share similar attitudes, and non-academics may use inclusive language more than academics in certain settings. These results underscore the importance of addressing gender stereotypes and implementing policies and training to promote gender equality in everyday communication. Future pathways for research are discussed.

## 1 Introduction

The use of gender-inclusive language is a highly contested field, even though findings on the relevance, effectiveness and feasibility of gender-fair language, collected by now over decades and for a broad range of languages,^1^ are rather clear: traditional generic masculine forms result in male biased mental representations and go along with decisions that disadvantage women (Stahlberg et al., 2007). Gender-fair forms have been shown to mitigate these tendencies and to constitute no linguistic barrier for individuals, regardless of their educational background (Friedrich and Heise, 2019; Pabst and Kollmayer, 2023; Renström et al., 2022); however, some studies indicate only a partial mitigating effect (e.g., Abbondanza et al., 2025) or observed impaired comprehension in specific conditions (e.g., Friedrich et al., 2024). Despite the fact that the majority of findings indicate that gender-inclusive language is effective and feasible, gender-inclusive language use is far from common, and there are even policies that forbid the use of certain forms in schools or official correspondence.^2^ There has been an increasing number of studies in recent years of which factors make people more or less likely to use gender-inclusive language. However, findings so far remain unintegrated and thus clear conclusions difficult. The aim of the current review is to systematize and integrate studies on what influences gender-inclusive language use in order to inform future research and to provide a basis for evidence-based strategies that could shape policy making as well as training and education.

Gender-inclusive language (also referred to as gender-fair or gender-sensitive language) is defined as language that avoids the use of the generic masculine form. This can be achieved through using existing forms via the strategies of feminization, i.e., explicit mention of both women and men (e.g., in German *Lehrerinnen und Lehrer*; teachers_fem_ and teachers_masc_), or neutralization, i.e., using epicene nouns (*Lehrkräfte*, teachers) or forms that are grammatically not gender marked (*Lehrende*, teaching persons), and their context-dependent use (Cacciari et al., 2011; Gabriel et al., 2018; Irmen and Roßberg, 2004). Recent definitions increasingly incorporate the inclusion of individuals outside the binary gender system, which can be, again, realized using existing gender-neutral forms, or via using newly created forms, like the gender asterisk (Lehrer*innen, teachers_masc*fem_) or the colon (*Lehrer:innen*, teachers_masc:fem_; Kolek, 2019; Zacharski and Ferstl, 2023). Avoiding the generic masculine form aims to ensure that all addressed individuals are equally represented in terms of gender. This issue is particularly relevant in languages with grammatical gender, such as French or Polish, where every noun is assigned a grammatical gender (feminine or masculine in French, feminine, masculine or neuter in Polish), and dependent forms such as articles or adjectives must agree with this gender. In languages with grammatical gender the generic masculine is often used as a supposedly neutral default for all genders (such as French *Tous les enseignants étaient arrivés*. All the teachers_masc_plural_ had arrived.). In languages with natural gender (e.g., English or Danish), most nouns do not belong to a grammatical gender category and avoidance of masculine generics mainly refers to gender-inclusive pronouns (e.g., A student must do their/his or her homework). In so called genderless languages (e.g., Finnish, Turkish), neither nouns nor pronouns are formally marked for gender, and gender is only assigned based on a noun’s meaning (Corbett, 1991; Stahlberg et al., 2007). In natural gender languages (and obviously in genderless languages) the generic masculine is less prominent than in grammatical gender languages, allowing for an easier implementation of gender-inclusive language. Beside these formal characteristics, using gender-inclusive language also entails communicating without reinforcing gender stereotypes (e.g., stereotypical role distributions such as in German *die Krankenschwester*_fem_ *und der Chefarzt_masc_*; the nurse and the chief physician; Sczesny et al., 2016). Thus, both aspects—the formal linguistic features and the conceptual level of linguistic discrimination—are encompassed in the term gender-inclusive language.

Sexist language, by contrast, is defined by Parks and Roberton (1998), p. 455 as including “words, phrases, and expressions that unnecessarily differentiate between women and men or exclude, trivialize, or diminish either gender.” According to this definition, sexist language reinforces gender stereotypes and perpetuates discrimination. Additionally, and as described above, formal linguistic structures—such as the use of the generic masculine—create asymmetries that further reinforce existing gender disparities as shown for many languages (Hellinger and Bußmann, 2001).

Effects of male biased cognitive representations on thinking and behavior have been shown in numerous studies. For example, masculine personal pronouns in mock job interviews resulted in a lower sense of belonging, motivation and identification with a job among female participants (Stout and Dasgupta, 2011). Potential female applicants for a high-status position perceived themselves as less suitable than male applicants when the job title was phrased in the masculine form. When the feminine form was included alongside the masculine, this effect disappeared (Horvath and Sczesny, 2016). This effect, although less pronounced, is also evident in men’s reactions to female occupational titles (Bem and Bem, 1973), indicating that it is not a gender-specific effect. However, due to the generic use of masculine forms, men are naturally affected to a much lesser extent.

Even girls of primary school age showed lower interest in typically male professions (e.g., astronaut, firefighter) and had lower confidence in being able to take them up when these jobs were described to them in generic masculine rather than in pair forms (Vervecken and Hannover, 2015).

To summarize, research findings clearly demonstrate that the use of gender-inclusive language is useful, effective and possible. Given the substantial evidence, the question arises why resistance persists and what factors influence it. From discussions in academia and media in the 1970s, Blaubergs (1980) identified eight distinct categories in which the prevailing arguments against gender-inclusive language could be classified: 1. “cross-cultural” arguments, which cite countries where language may be more gender-fair, yet gender equality is less advanced than, for instance, in English-speaking nations, 2. “language is a trivial concern” arguments, 3. “freedom of speech” arguments, 4. “sexist language is not sexist” arguments, 5. “word-etymology” arguments referring to the original, often generic, meaning of words (e.g., man), 6. “appeal to authority” (such as linguists) arguments, 7. “change is too difficult” arguments, and 8. “it would destroy historical authenticity and literary work” arguments. As subsequent studies show, these categories are still relevant. In a study on attitudes toward gender-inclusive language, Parks and Roberton (1998) found that 40% of respondents named the difficulties associated with change as their primary reason for rejecting gender-inclusive language, and 11% stated that sexist language is not sexist. Vergoossen et al. (2020) again identified categories similar to Blaubergs’ within broader dimensions, such as acceptance of sexism or cisgenderism, and the belief that generic formulations are not sexist. Approximately 40% of statements made by participants in this study were categorized as emphasizing the traditional use of language, including the belief that linguistic change is too difficult. Interestingly, only a small proportion of participants, 6.3%, found gender-inclusive language to be disruptive to communication.

Several studies show that attitudes toward gender-inclusive language are closely related to actual language use. This was shown, for example, in a German study employing Ajzen’s theory of planned behavior (Ajzen, 1991; Sczesny et al., 2015). In the study’s first part, participants completed a questionnaire assessing, among others, their attitudes toward gender-inclusive language and intentions regarding its use. Additionally, they provided self-report data on the frequency of their use of gender-inclusive language. Two weeks later, participants’ actual use of gender-inclusive language was measured using a sentence completion task, in which they had to choose between a generic masculine or a gender-inclusive form. The results showed that the intention to use gender-inclusive language was strongly associated with positive attitudes. Actual language use was moderately correlated with both intention and gender-inclusive attitudes. In a similar study, Gustafsson Sendén et al. (2015) reported an even stronger correlation between attitudes and self-reported linguistic behavior in users of Swedish. Even though self-reports may be more prone to biases than behavioral data, empirical evidence indicates that – in line with the theoretical framework – there is a systematic relation between attitudes and behavior with regard to gender-inclusive language, something which underlines the usefulness of including studies on attitudes and those on behavior in our review.

The aim of this review is to identify relevant factors from the literature that have been shown to influence attitudes toward and use of gender-inclusive language, and to provide an integrated analysis from which evidence-based research and policy strategies can be derived.

## 2 Method and structure

To achieve our research goal, we searched for empirical studies on attitudes toward and use of gender-inclusive language in relevant databases (PsychInfo, Psyndex, and Google Scholar). The search was conducted between March and July 2024 as part of the first author’s bachelor’s thesis (Lange, 2024), on which parts of this manuscript are based, and repeated in March 2025. Our search terms contained the combinations of “influence of” with “sex”, “gender”, “age”, “time”, “exposure duration”, “situation”, “education”, “educational level/background”, “personality”, “social relationships”, “sexism”, “attitudes toward women”, “cisgenderism”, “empathy”, “society”, “culture”, “political orientation”, “religion”, “religious beliefs”, “tradition”, “traditional values”, “motivation”, “interest”, and “language proficiency” “on attitudes toward/the use of” “gender-fair/gender-inclusive/sexist language”. We also looked for similar studies, for example, using Google Scholar’s “cited by” feature. We excluded studies that did not collect original data. From our search we retrieved 34 studies in diverse languages both with and without grammatical gender systems, and with varied research methods comprising subjective measures, behavioral measures in experimental designs and intervention studies, and corpus analyses. This linguistic and methodological breadth allows us to draw conclusions irrespective of a specific language, method or design. The influencing factors that were investigated in the reviewed studies reflect key research foci in the field, whose relevance is founded both theoretically and empirically. The sequence of our presentation reflects the breadth of the database that could be found regarding a respective factor proceeding from broader to smaller. For each factor, we first discuss all identified studies per factor on attitudes followed by all identified studies on language use. Attitudes are conceptualized, in general and in the reviewed studies, as relatively enduring evaluations of gender-inclusive language which are tied to societal beliefs and ideologies, and which can evolve and are based on emotional, affective, and cognitive aspects. After presenting each factor separately, we provide an integrative discussion and conclusion. All studies covered in this review and the factors of influence can be found in Table 1. Some studies include data on more than one factor (e.g., gender and age; Gustafsson Sendén et al., 2015; Rubin and Greene, 1991). Therefore, these studies occur at several points in our review and in more than one section of Table 1. Factors with a limited number of studies (1 or 2) are briefly described in section 3.9.

**Table 1:** Overview of reviewed factors and corresponding studies (total *k* = 34).

Factor	Attitude	Use
Sex and gender (*k* = 17)	Bruns and Leiting (2024), Bruns and Leiting (2025), Gustafsson Sendén et al. (2015), Parks and Roberton (2004), Parks and Roberton (2005), Remigio and Talosa (2021), Renström et al. (2022), Rothmund and Christmann (2002), Rubin and Greene (1991), Sczesny et al. (2015), Steiger and Irmen (2011), and Talosa (2018), (*k* = 12)	Bruns and Leiting (2024), Gustafsson Sendén et al. (2015), Jacobson and Insko (1985), Koeser et al. (2015), Lee (2007), Renström et al. (2022), Rubin et al. (1994), Sczesny et al. (2015), and Steiger and Irmen (2007), (*k* = 9)
Age and exposure duration (*k* = 11)	Bruns and Leiting (2024), Bruns and Leiting (2025), Gustafsson Sendén et al. (2015), Gustafsson Sendén et al. (2021), Parks and Roberton (2005), Parks and Roberton (2008), Renström et al. (2022), Rubin and Greene (1991), and Talosa (2018), (*k* = 9)	Bruns and Leiting (2024), Gustafsson Sendén et al. (2015), Gustafsson Sendén et al. (2021), Renström et al. (2022), Rubin et al. (1994), and Steiger and Irmen (2011), (*k* = 6)
Sexism (*k* = 8)	Gustafsson Sendén et al. (2015), Parks and Roberton (2004), Parks and Roberton (2005), Sarrasin et al. (2012), and Sczesny et al. (2015), (*k* = 5)	Cralley and Ruscher (2005), Gustafsson Sendén et al. (2015), Jacobson and Insko (1985), and Swim et al. (2004), (*k* = 4)
Social and political influence (*k* = 8)	Formanowicz et al. (2015), Sarrasin et al. (2012), and Strafelda (2018), (*k* = 3)	Gustafsson Sendén et al. (2015), Gustafsson Sendén et al. (2021), Hodel et al. (2017), Link (2024), and Waldendorf (2024), (*k* = 5)
Political orientation (*k* = 7)	Formanowicz et al. (2015), Gustafsson Sendén et al. (2015), Gustafsson Sendén et al. (2021), and Renström et al. (2022), (*k* = 4)	Gustafsson Sendén et al. (2015), Gustafsson Sendén et al. (2021), Renström and Klysing (2024), Stecker et al. (2021), and Waldendorf (2024), (*k* = 5)
Situational context (*k* = 5)	Koeser and Sczesny (2014), Parks and Roberton (2002), and Rubin and Greene (1991), (*k* = 3)	Koeser and Sczesny (2014), Koeser et al. (2015), and Rubin et al. (1994), (*k* = 3)
Interest in gender related topics (*k* = 3)	Gustafsson Sendén et al. (2015), Gustafsson Sendén et al. (2021), and Renström et al. (2022), (*k* = 3)	Gustafsson Sendén et al. (2015) and Gustafsson Sendén et al. (2021), (*k* = 2)
Educational level (*k* = 3)	Pabst and Kollmayer (2023) and Steiger and Irmen (2011), (*k* = 2)	Kuhn and Gabriel (2014) and Steiger and Irmen (2011), (*k* = 2)
Influences with small database		
Personality (*k* = 2)	Parks and Roberton (2005) and McMinn et al. (1990)	
Motivation for accuracy in language use (*k* = 1)	Kuhn and Gabriel (2014)	
Traditionalism and Cisgenderism (*k* = 1)	Renström et al. (2022)	
Religious orientation (*k* = 1)	McMinn et al. (1990)	

Several studies investigated more than one factor and therefore are listed repeatedly.

## 3 Empirical evidence on possible factors influencing attitudes toward and use of gender-inclusive language

### 3.1 Sex, gender, and gender identity

The influence of sex, gender and, more recently, gender identity, has found the most attention in the research field (see Table 1) and was investigated in 17 studies, 12 of which assessed effects on attitudes toward gender-inclusive language, and 9 assessed effects on language use. In the identified studies, either sex or gender was used as a predictor, though particularly in older work, both terms often referred predominantly to biological sex. In some more recent studies gender identity was also used as a predictor of gender-inclusive language attitudes and use. Since sexist language primarily disadvantages women and non-binary individuals, a gender-based comparison of attitudes and behaviors toward gender-inclusive language is both relevant and insightful. According to Social Identity Theory (Tajfel and Turner, 1979, 1986), women and non-binary people can be expected to hold more favorable attitudes toward gender-inclusive language and to use it more frequently than men, as they identify more strongly with those negatively affected by linguistic sexism.

#### 3.1.1 Attitudes

Rubin and Greene (1991) examined attitudes toward gender-inclusive language in an interview setting and recorded participants’ gender and age. The interview questionnaire was adapted from Henley and Dragun (1983) and included six components; one of which was the level of concern—a measure assessing whether the respondents actually thought about sexism in language, considered it a problem, and actively tried to avoid sexist formulations. A higher number of male participants reported the lowest level of concern, while women rated sexist language as more problematic and identified more ways to avoid it. Rothmund and Christmann (2002) compared the reception of short texts, which were phrased either in different versions of gender-fair formulations or with masculine generic forms; they assessed their perceived simplicity and readability, conciseness, motivational stimulation and esthetics, and participants’ attitudes toward gender-inclusive language. While text versions did not differ in their perceived communicational qualities, women expressed more positive attitudes toward gender-inclusive language compared to men. Parks and Roberton (2004, 2005) focused on attitudinal differences between men and women toward sexist language. They developed the Inventory of Attitudes toward Sexist/Nonsexist Language – General (IASNL-G; Parks and Roberton, 2000, 2001), which evaluates attitudes in three distinct domains: 1. willingness to use inclusive language, 2. ability to correctly identify sexist language, and 3. beliefs about sexist language, such as whether it should be removed from common usage. Their findings showed that women exhibited more positive attitudes toward gender-inclusive language than men. However, while women’s mean scores fell within the neutral range, men’s attitudes varied at slightly above neutral (Parks and Roberton, 2004) or slightly below neutral (Parks and Roberton, 2005). Similar findings have been reported in Swedish-language studies. In 2015, Sweden officially introduced the gender-neutral pronoun *hen* into dictionaries (e.g., Svenska Akademien, 2015) as a step toward gender-inclusive language. *Hen*, which has no gender marking, can be used either generically, when the gender of a referent is irrelevant, or specifically, to refer to non-binary individuals (Gustafsson Sendén et al., 2015). The introduction of *hen* prompted researchers to examine potential shifts in attitudes and behavior over time, as well as gender differences in these attitudes. In their study, Gustafsson Sendén et al. (2015) did not distinguish between the generic and specific uses of *hen*, whereas Renström et al. (2022) separately assessed participants’ attitudes toward each function. Regardless of this distinction, women consistently expressed more positive attitudes toward the pronoun than men. Both studies also measured participants’ gender (role) identity, i.e., the extent to which individuals define themselves based on traditionally feminine or masculine traits. This was assessed using items adapted from Luhtanen and Crocker’s (1992) collective self-esteem scale, which examines the psychological importance of gender identity. While Renström et al. (2022) found no significant relationship between gender identity strength and attitudes toward *hen*, Gustafsson Sendén et al. (2015) reported that gender identity strength was an even stronger predictor of attitudes than biological sex. The influence of biological sex was comparable across both studies, but the effect of gender identity differed despite the shared linguistic and political context and similar survey instruments. However, sampling methods and demographics may have contributed to that. Gustafsson Sendén et al. (2015) recruited participants through a combination of methods, including students, passersby in train stations and town centers (using paper-and-pencil surveys), as well as online respondents. Their sample had an average age of 32 years. In contrast, Renström et al. (2022) used an independent survey research company, which generated a sample with an average age of 50 years.

In the previously mentioned German-language study by Sczesny et al. (2015) participants reported neutral attitudes toward gender-inclusive language on average, regardless of gender. This result is particularly striking because the sample was drawn from an online survey, similar to studies in Sweden, where gender differences were observed. Additionally, the attitude measurement items used in the study closely resembled those employed by Rubin and Greene (1991), which also identified gender effects. Similar inconsistencies can be found in further English-language studies. Talosa (2018) and Remigio and Talosa (2021) examined the attitudes of male and female university students toward gender-inclusive language and found no significant differences between them. Notably, both studies utilized the IASNL-G scale (Parks and Roberton, 2000), which was also employed in studies that did detect gender-based differences (Parks and Roberton, 2004, 2005). Further variations emerge when examining specific samples. In a German-language study by Steiger and Irmen (2011), attitudes toward gender-inclusive wording were investigated in three distinct samples: vocational trainees without a school graduation, individuals aged 60 and older, and legal professionals. The male and female participants in the trainee and 60 + samples did not differ in their attitudes. However, within the legal professional sample, female participants were more accepting of gender-exclusive language (i.e., the generic masculine) than their male colleagues. Bruns and Leiting (2024, 2025) assessed attitudes toward newly created forms of gender-inclusive language (e.g., the asterisk) and existing forms (e.g., epicene nouns) in a closed and an open response format and found that non-cisgender participants held more favorable attitudes than both women and men who in turn did not differ from each other. They also found that in the open format data non-cisgender participants named more arguments and a greater variety of reasons for their stance than women who in turn named more arguments and more various reasons than men.

#### 3.1.2 Language use

Jacobson and Insko (1985) let participants complete text gaps based on their linguistic preferences, choosing from five alternative formulations (*he, she, he/she,* and two item-specific alternatives). The sentences focused on stereotypically male and female professions, as well as professions ending in -*man* (e.g., spokesman), which at the time of the study were often used generically. Results indicated that women used the gender-inclusive formulation *he/she* more often in masculine sentences than men did, whereas men were more likely to choose *he* in both masculine and generic contexts. The study also found that gender influenced the use of *she*: while men applied the feminine form more often in feminine-associated sentences, women were more likely to use it in masculine contexts. Similar findings were observed in other English-language studies. Rubin et al. (1994) found that men more frequently chose non-inclusive formulations such as -*man* or the generic masculine—in fact, men used the generic masculine more than four times as often as women did. Their study also distinguished between eight types of gender-inclusive alternatives, namely: 1. replacements for the generic masculine (e.g., humankind instead of mankind), 2. singular they/them, 3. the use of passive voice, 4. the use of *one*, 5. continuous use of singular noun (e.g., the student instead of he or she), 6. the paired form (he/she), 7. combining masculine and feminine terms (boys and girls), and 8. pluralization (students). Women were found to use pluralization and *one* more frequently than men, while men favored the other alternatives.

Gender differences have also been reported regarding the use of the gender-neutral pronoun *hen* (Gustafsson Sendén et al., 2015; Renström et al., 2022). In both studies, women reported using *hen* more frequently than men. However, the effect of gender lost statistical significance when other factors—such as sexism, cisgenderism, or general interest in gender-related issues—were accounted for. In a German-language experimental study, Koeser et al. (2015) investigated whether reading different text conditions (gender-inclusive language, generic masculine forms, avoiding person reference, for example via passive voice, topic without person references) could affect participants’ subsequent use of gender-inclusive forms in a sentence completion task. Although overall gender-inclusive formulations were used infrequently (on average, in only 2–3 out of 10 gaps), women who had previously read a gender-inclusive text were more likely to use gender-inclusive language than men in the same condition. Women used fewer gender-inclusive wordings in all other text conditions, whereas men’s language use remained unchanged regardless of text condition. Lee (2007) examined Cantonese-speaking participants translating seven occupational titles into English. While four out of seven translations (e.g., police officer, firefighter, post officer, and salesperson) showed no gender differences, women were more likely to translate chairperson and spokesperson using gender-neutral forms, whereas men did so more frequently for businessperson. Further complicating the picture, some studies report no gender effects at all. Sczesny et al. (2015) and Steiger and Irmen (2007), in their respective German-language studies, found no gender differences in their sentence completion tasks. Bruns and Leiting (2024) assessed self-reported as well as actual use of several existing and newly created forms of gender-inclusive language. For measuring the use they employed a translation task where a sentence that contained a singular indefinite referent and a pronoun that referred to that person had to be translated from English to German (e.g., *A writer must find their own style of writing to be successful*.). Highest self-reported use as well as highest actual use of gender-inclusive language in the translation task was found in non-cisgender participants, followed by women, followed by men.

#### 3.1.3 Summary

In summary, gender per se does not appear to reliably predict attitudes or behavior regarding gender-inclusive language. When differences are found (12 out of 17 studies), women tend to express more positive attitudes (6/12) and use gender-inclusive wording more frequently than men (6/9), which is consistent with predictions based on the theory of social identity (Tajfel and Turner, 1979, 1986). However, women’s attitudes tend to be neutral rather than positive, and gender-inclusive formulations remain infrequent overall. Attitudes and behavior appear to be influenced by specific conditions, highlighting the situational and contextual nature of linguistic behavior. Furthermore, some studies indicate that the predictive power of gender diminishes – or even disappears – when overarching ideologies such as sexism or cisgenderism are considered. Accordingly, future research should shift its focus from gender as a primary variable to more nuanced sociocultural and ideological factors, which seem to play a more substantial role in shaping linguistic attitudes and behavior. Including non-binary individuals is also crucial, as they have been underrepresented in previous research, yet offer valuable and enriching perspectives – particularly in the context of cisgenderism.

### 3.2 Age and exposure duration

The mere-exposure effect (Zajonc, 1968) suggests that repeated exposure improves attitudes toward a stimulus. Applied to gender-inclusive language, this implies that increased exposure could foster more positive attitudes. Consequently, one might hypothesize that older individuals, having encountered gender-inclusive language more frequently throughout their lives, would exhibit more positive views. However, they have also had more exposure to non-inclusive linguistic forms. Therefore, it is crucial to investigate the relationship between age, exposure duration to gender-inclusive formulations, and corresponding attitudes or behaviors. We identified 11 studies in this category, 9 examining effects on attitudes and 6 assessing effects on the use of gender-inclusive language.

#### 3.2.1 Attitudes

An early comparison between age groups regarding attitudes toward gender-inclusive language in English-speaking contexts was made by Rubin and Greene (1991) who compared students aged 18 to 25 with individuals aged 30 to 45 who had completed higher education. The comparison was based on responses to interview questions that determined whether avoiding sexist language was an important concern for a person and whether they actively tried to eliminate it from their speech. Results showed that three times as many older participants as younger ones reported the highest level of concern. Participants were also asked to evaluate how sexist they perceived the English language. They also had to indicate whether they sought gender-inclusive alternatives, which methods they used, and what their motives were. Although older participants reported using more methods to avoid sexist language than younger ones, both age groups rated the level of sexism in language similarly and avoided it for similar reasons. Parks and Roberton (2005) examined attitudes toward gender-inclusive language in two age groups (18–19 years and 21–23 years) using their IASNL-G scale. Even between these two closely related age groups, there were slight but significant differences in attitudes, with older participants displaying more positive attitudes than younger ones. When the data was separated by gender (male, female), the effect of age remained significant only for men. In a subsequent study older age groups were included (18–22 years, 30–49 years, 51–69 years, and 70–87 years; Parks and Roberton, 2008). Here, the youngest group held more negative attitudes than almost all other age groups. Only the 70- to 87-year old participants did not differ from the youngest group. Despite the significant differences, the mean values for both younger and older participants remained within the range of neutral attitudes. Talosa (2018) studied a relatively young sample with 90.47% of participants between 17 and 22 years old (students in teacher education at a Philippine university) employing the IASNL-G. Older participants (21+) were better at identifying sexist language than younger ones, but there was no effect of age on “willingness to use inclusive language” and “beliefs about sexist language”. Renström et al. (2022) examined attitudes toward the personal pronoun *hen* and found more positive attitudes in younger compared to older participants. This reflects an effect of age without the aspect of exposure since the pronoun was newly introduced and unfamiliar to all age groups. Interestingly, age remained a significant predictor even when additional predictors (e.g., interest in gender issues) were included in the analysis.

Gustafsson Sendén et al. (2015) examined changes in attitudes toward *hen* over time. The proportion of individuals with a negative attitude dropped significantly from 56.5% in 2012 to 9.6% in 2015, while the proportion of those with a positive attitude increased substantially from 17.4 to 68.9% over the same period. Younger participants expressed more positive attitudes than older ones. A follow-up study (Gustafsson Sendén et al., 2021) found a similar positive trend in attitudes between 2015 and 2018. Once again, younger participants’ attitudes were more positive compared to older ones. Moreover, the change of attitudes over time varied with age: younger participants’ attitudes became more positive over the three-year period, whereas those of older participants remained negative. In the study by Bruns and Leiting (2024, 2025) younger and older participants (17–27 years, 45–80 years, respectively) did not vary in their overall attitudes; however, regarding the reasons they named for their attitude toward existing and newly created gender-inclusive language forms, younger people more often mentioned inclusivity, and older ones clarity of rules and esthetics.

#### 3.2.2 Language use

Rubin et al. (1994) analyzed language use in English speeches by male business leaders over time. They found a significant decrease in frequency of masculine generic formulations from the 1960s to the 1970s and 1980s (which in turn did not differ from each other). In their first study on the newly introduced pronoun *hen* Gustafsson Sendén et al. (2015) found that younger participants reported using *hen* more often than older participants. However, in a follow-up study (Gustafsson Sendén et al., 2021) it was the older participants who reported more frequent usage. Renström et al. (2022) employed a gap-fill task and did not find a clear relation between participants’ age and their use of *hen*. Steiger and Irmen (2011) also used a gap-fill task to measure gender-inclusive language use in German. When participants could select from various alternative expressions, a masculine generic and several gender-inclusive versions, language use differed with age. Participants aged 60 to 85 avoided generic masculine forms to complete the gaps, whereas younger participants (aged 16 to 42) tended to use them more often (but still less often than alternative forms). Bruns and Leiting (2024) found that in a translation task from English to German both young and older participants used mostly generic masculine translations, but differences occurred in strategies regarding gender-inclusive language. Younger participants used more newly created, or what the authors called visible inclusive forms including gender asterisk or colon (e.g., der*die, the_masc_*the_fem_; *Lehrer:innen*, teachers_masc:fem_) whereas older participants used more existing lexical forms such as participles, epicenes or avoidance of a role noun through describing the activity (*Lehrende, Lehrkraft, Person, die lehrt*, i.e., teaching person, teacher/teaching staff, person who teaches).

#### 3.2.3 Summary

In summary, regarding age, it appears that the type of gender-inclusive language that is being examined makes a difference—whether it is language change via a new pronoun or inclusive forms with word internal symbols versus more general non-sexist formulations. Older individuals seem to find it easier than younger ones to recognize the negative impact of sexist language in everyday life but are less inclined to adopt newly introduced forms. Younger people may adapt more easily to new forms than older people, who have been exposed to traditional language structures for a longer time. Of particular interest would therefore be a comparison between age groups regarding attitudes toward gender-inclusive language in general and new, specific gender-inclusive forms, such as the pronoun *hen* or the gender asterisk. Similar to sex and gender, age alone appears to provide little conclusive insight into individuals’ attitudes or language use. The only consistently observed effect is that exposure time—such as to *hen*—correlates positively with both positive attitudes (2 of 2 studies, which considered exposure duration) and inclusive language use (3/3), thereby indicating a mere-exposure effect.

### 3.3 Sexism

Acceptance of sexism has been identified as a relevant category in studies on gender-inclusive language (Parks and Roberton, 1998; Vergoossen et al., 2020) and manifests in prejudiced attitudes and discriminatory behaviors toward individuals based on their gender. Due to social pressure, sexism is no longer as openly communicated as in the past but is instead expressed in more subtle ways as captured in the concepts of modern sexism (Swim et al., 1995), ambivalent sexism (Glick and Fiske, 1996), and neosexism (Tougas et al., 1995). Modern sexism becomes evident in statements that deny the ongoing discrimination against women, ambivalent sexism reinforces gender stereotypes in either a hostile or seemingly benevolent manner, while neosexism captures the conflict between egalitarian values and negative attitudes toward women. Based on their respective definitions, each of these forms of sexism is assumed to be associated with a rejecting attitude toward gender-inclusive language and its use: modern sexism, as it denies the need for linguistic change based on the belief that gender equality has already been achieved; ambivalent sexism, as it accepts and even reinforces gender inequality; and neosexism, as prejudices against women persist despite a professed liberal value system and may also be expressed linguistically. We identified 8 studies on this factor, 5 examining effects on attitudes toward gender-inclusive language, and 4 investigating effects on language use.

#### 3.3.1 Attitudes

Parks and Roberton (2004) investigated sexism as a potential mediating factor in the relationship between a person’s gender and their attitudes toward gender-inclusive language. Three different questionnaires were used to assess sexism: one measuring overt sexism (Attitudes Toward Women Scale, Spence and Hahn, 1997) and two measuring subtle forms of sexism (Modern Sexism Scale, Swim et al., 1995; Swim and Cohen, 1997; Neosexism Scale, Tougas et al., 1995). Attitudes were assessed using the IASNL-G (Parks and Roberton, 2000). The results showed that all three sexism measures were negatively correlated with attitudes toward gender-inclusive language. This result was replicated in a subsequent study by Parks and Roberton (2005), where sexism was measured using the Neosexism Scale (Tougas et al., 1995). Sarrasin et al. (2012) examined the relationship between different forms of sexism (modern, hostile, benevolent) and attitudes toward gender-inclusive language (Language Use Questionnaire, Prentice, 1994; IASNL-G’s subscale on the correct identification of sexist language, Parks and Roberton, 2000) in various linguistic and political contexts. The study surveyed German-speaking and French-speaking students in Switzerland as well as English-speaking students in the United Kingdom. Attitudes toward gender-related language reforms were negatively correlated with modern and hostile sexism whereas benevolent sexism had no effect. Regarding the correct identification of sexist language, only modern sexism was found to have a significant impact, making it more difficult for those with high modern sexism scores to recognize sexist expressions. The German-language study by Sczesny et al. (2015) measured modern sexism, ambivalent sexism, and neosexism. Similar to Sarrasin et al. (2012), a negative relationship between modern sexism and attitudes toward gender-inclusive language was found. Additionally, this relationship was observed for both other forms of sexism, with the effect of ambivalent sexism (across both hostile and benevolent forms) being the weakest. Similar results were also found in a study on the Swedish gender-neutral pronoun *hen*, where respondents’ levels of modern sexism were negatively correlated with their attitudes toward *hen* (Gustafsson Sendén et al., 2015).

#### 3.3.2 Language use

Jacobson and Insko (1985) let participants complete cloze tests with five alternative pronoun choices (he, she, he/she, and two additional context-specific options). Sexism was assessed using the Attitudes Toward Women Scale (Spence and Helmreich, 1972). As expected, participants with higher sexism scores used the generic masculine pronoun *he* more frequently than those with lower sexism. A similar study by Swim et al. (2004) examined overt sexism (Spence et al., 1973), modern sexism, and ambivalent sexism. Participants were asked to respond to three moral dilemmas in short written texts, describing the actions of protagonists in stereotypically gendered professions (business executive, nurse, professor). The texts were analyzed for gender-exclusive (e.g., *she* for the nurse, *he* for the other professions) and gender-inclusive formulations (e.g., *he or she* in all cases). Modern sexism emerged as the strongest predictor of language use, with sexism scores positively correlating with the use of non-inclusive expressions. Cralley and Ruscher (2005) examined whether men’s level of modern sexism predicted their use of sexist language when describing images of women in neutral contexts. In both written and spoken descriptions, men with higher sexism scores used more sexist terms (e.g., *girl* or *babe*) than those with lower sexism. However, when cognitive load was increased, no differences between high-sexism and low-sexism individuals were observed. Also, in the Swedish-language study by Gustafsson Sendén et al. (2015), a negative correlation was found between self-reported use of the gender-neutral pronoun *hen* and levels of sexism, measured by the Swedish version of the Modern Sexism Scale (Ekehammar et al., 2000).

#### 3.3.3 Summary

In summary, the empirical evidence indicates that – as predicted – sexism is a strong predictor of both attitudes toward (5/5) and use (4/4) of gender-inclusive language with modern sexism showing the most consistent effects across different linguistic contexts. As modern sexism reflects the belief that gender equality has already been achieved, these findings underscore the ongoing need to raise awareness of persistent gender inequalities and their consequences. Moreover, the evidence that increased cognitive load leads to reduced use of gender-inclusive language and a reversion to habitual sexist expressions highlights the importance of practice and supportive regulations.

### 3.4 Social and political influence

The findings on the impact of exposure duration to new linguistic wording discussed in section 3.2 could also reflect social and political influences. The influence of social groups (e.g., political parties) on members’ thoughts and actions via established norms can be linked to Social Identity Theory (Tajfel and Turner, 1979, 1986), where individuals internalize ingroup values and attitudes. According to the Theory of Planned Behavior (Ajzen, 1991), these internalized attitudes are then expected to translate into behavioral intentions, followed by actual behavior. We identified 8 studies in this category, 3 examining effects on attitudes toward gender-inclusive language (2 regarding effects of societal changes, 1 regarding effects of close relationships) and 5 investigating effects on language use.

#### 3.4.1 Attitudes

Sarrasin et al. (2012) compared attitudes toward gender-inclusive language in the United Kingdom with those in German-speaking and French-speaking regions of Switzerland. While the UK took political action against gender discrimination relatively early (e.g., the *Sex Discrimination Act* of 1975), similar legal measures emerged in Switzerland later (e.g., the *Equality Act* of 1996; Sarrasin et al., 2012). Using a subscale of the IASNL-G, participants’ recognition of sexist language was compared between English and Swiss (German-speaking and French-speaking) participants. Results showed higher scores for English than Swiss participants, something which may reflect socio-political differences between the countries but could also reflect effects of grammatical gender with English being a natural gender language and French and German both having a grammatical gender system. Thus, effects due to country and due to grammatical gender cannot be separated.

In an experimental study, Formanowicz et al. (2015) compared German speakers in Austria, where gender-inclusive language is widely implemented and required in job advertisements, and Polish speakers in Poland, where such language is relatively new. Both languages have a grammatical gender system. Participants rated a text describing a social initiative regarding female professional groups which were described using either the traditional generic masculine or a feminine form. The results showed that Polish participants rated the text more positively when written in the generic masculine, whereas Austrian participants responded more favorably to the feminine version.

Beyond societal-level influences, an individual’s immediate social environment can also shape their attitudes, a phenomenon consistent with Heider’s (1946) Balance Theory, which postulates that people strive for cognitive balance in their relationships and attitudes and therefore may align their own beliefs. Strafelda (2018) examined whether close relationships with female persons influence attitudes toward gender-inclusive language. This study also used the IASNL-G scale and included yes/no questions about the presence of female family members and other close contacts (e.g., “Do you have any sisters (including biological, step, half, and adopted)?”). However, no significant results were found, something which the author attributes to limiting factors, including the binary response format, which may not have adequately captured the degree of influence, and the lack of information on whether the influence was perceived as positive or negative. Beyond this study, we found no further research on the influence of close contacts on attitudes toward gender-inclusive language. However, numerous studies confirm the impact of core family structures on attitudes (e.g., on adolescent sexism; Dueñas et al., 2020), suggesting that close relationships could also shape attitudes toward gender-inclusive language.

#### 3.4.2 Language use

Gustafsson Sendén et al. (2015, 2021) showed that over the years the proportion of individuals who do not use gender-neutral *hen* decreased. Studies that compare gender-inclusive language use between nations and languages also reflect the influence of political factors. For example, Hodel et al. (2017) compared job advertisements in two Slavic-speaking countries (Poland, Czech Republic) with two German-speaking countries (Switzerland, Austria). While all languages, Polish, Czech, and German have grammatical gender systems, the countries rank differently in their societal level of gender equality (measured by the Global Gender Gap Index; Hausmann et al., 2012). Results show that job postings in the German-speaking countries were more likely to employ gender-inclusive wording than those in Slavic-speaking countries. In a diachronic study of newspaper articles, Waldendorf (2024) showed that gender-inclusive language is now used far more frequently in national German newspapers across the political spectrum than in the past; this shift is attributable to evolving social norms as reflected in editorial guidelines. Left-leaning media showed a higher increase and used more non-binary inclusive forms (e.g., Forscher*innen, researchers_masc*fem_). Analyzing differences between Austrian, German and Swiss media in the use of gender-inclusive language, Link (2024) selected two newspapers per country, one from the left/liberal spectrum and one from the right/conservative spectrum, and found the most frequent use of gender-inclusive language (newly created and existing forms) in Austria, followed by Switzerland, and then by Germany. Moreover, there was a substantial increase in gender-fair forms in all three countries from 2017 onward, and a stagnation of this trend for the conservative Swiss newspaper and a decrease for both German newspapers from 2021 on. As mentioned before, these socio-political influences cannot be separated from the effect of time.

#### 3.4.3 Summary

In line with the theoretical foundation, both attitudes toward (2 of 2 studies, which focused on societal changes) and the use of (5/5) gender-inclusive language appear to change when social norms shift and values are redefined. Existing findings provide evidence for influences through gender equality legislation as well as through smaller scale policies, such as editorial regulations in media organizations. International comparisons suggest that both evolving social norms and political measures can contribute to the promotion of gender-inclusive language. Further research is needed to clarify whether and how close personal relationships influence attitudes and behaviors regarding gender-inclusive language.

### 3.5 Political orientation

As mentioned before, membership in a political party (as a social group), can shape the members’ attitudes and behaviors. While political conservatives traditionally uphold values that support stereotypical gender roles, liberals tend to endorse universalistic and egalitarian values (Jones et al., 2018). It is therefore reasonable to assume that political orientation could affect the stance toward gender-inclusive language. We identified 7 studies in this category, 4 examining effects on attitudes toward gender-inclusive language and 5 assessing effects on language use.

#### 3.5.1 Attitudes

The study by Formanowicz et al. (2015) mentioned previously also examined the influence of political orientation, measured on a scale ranging from very liberal to very conservative, on evaluations of a text about a social initiative. For both German-speaking and Polish-speaking participants, the more conservative a person, the more negatively they evaluated the described initiative, irrespective of the used language. In contrast, the two studies by Gustafsson Sendén et al. (2015, 2021) on the neutral pronoun *hen* found a significant relationship between political orientation and attitudes toward the pronoun. The further right participants identified politically, the more negative their attitudes were toward *hen*. Interestingly, Renström et al. (2022) found political orientation to be predictive only when not accounting for additional factors, such as cisgenderism or preference for linguistic status quo. These variables were not measured in Gustafsson Sendén et al. (2015, 2021).

#### 3.5.2 Language use

Stecker et al. (2021) examined gender-inclusive language use in the German parliament (Bundestag) between 1949 and 2021 via the frequency of feminine occupational titles used by different parties (left-wing to right-wing: Die Linke [the left party], Die Grünen [the green party], SPD [Social Democrats], FDP [liberal democrats], CDU/CSU [Christian Democrats], and AfD [right-wing]). An increase in gender-inclusive language use was found since 1980, with more left and centre-left parties (Die Linke, Die Grünen and SPD) using it more frequently than centre-right (FDP) and conservative parties (CDU/CSU). In 2017, the contrast between political orientations became even more pronounced with the AfD joining the Bundestag, as this party used almost no feminine occupational terms. In Swedish studies examining the use of *hen* (Gustafsson Sendén et al., 2015, 2021), political orientation also emerged as a significant predictor of self-reported frequency of *hen* usage, with right-wing orientation being associated with less frequent use. Moreover, in an English-speaking study Renström and Klysing (2024) showed that higher values in right-wing authoritarianism (RWA; Bizumic and Duckitt, 2018) were related to less frequent use of singular they as a gender-neutral pronoun (in favor of gender specific pronouns). The study on language use in German newspapers by Waldendorf (2024) also suggests a relation between political orientation and frequency with more left-leaning outlets using more non-binary and binary gender-inclusive language.

#### 3.5.3 Summary

To summarize, political orientation has proven to be more consistent in predicting behavior (5/5) than attitudes (3/4). While a more liberal political orientation goes along with more frequent use of gender-inclusive language, a respective difference in attitudes was only found in studies on Swedish *hen*. Overall, research on this topic is limited, highlighting research gaps and the need for further investigation.

### 3.6 Situational context

In addition to stable factors and those developing in time, several studies examined the influence of more momentary conditions. We identified 5 studies in this category, 3 assessing effects on attitudes toward gender-inclusive language and 3 examining effects on language use.

#### 3.6.1 Attitudes

An experimental study by Rubin and Greene (1991) found that when interviewed about gender-inclusive language by a female, participants expressed significantly more critical views toward non-inclusive language and were more likely to perceive certain expressions as sexist. Koeser and Sczesny (2014) attempted to influence German-speaking participants toward more positive attitudes regarding gender-inclusive language through letting them read arguments supporting inclusive language, but without success. Similarly, an earlier intervention study using videos and audio messages to persuade participants to adopt more favorable attitudes toward gender-inclusive language did not yield significant effects either (Parks and Roberton, 2002).

#### 3.6.2 Language use

Rubin et al. (1994) asked participants to write a letter in response to a fictitious university-mandated drug test policy. The letter was to be addressed either to the university or to a friend. Additionally, participants writing the formal letter were instructed to adopt a convincing argumentative stance, whereas those writing to a friend were encouraged to express their personal feelings on the matter. The analysis revealed that participants, particularly men, used more inclusive expressions in the formal letter compared to the letter to a friend. Koeser et al. (2015) found that reading gender-inclusive texts led participants—especially women—to use gender-inclusive language more frequently. For men (but not for women), behavioral change only occurred when a direct reference to gender-inclusive language was stated in the text. Similar results were observed in a study where participants were exposed to arguments promoting gender-inclusive language (Koeser and Sczesny, 2014). Both strong arguments (e.g., gender-inclusive language as a crucial factor for gender equality) and weaker ones (e.g., existing evidence is ideology-free and therefore reliable) increased participants’ use of gender-inclusive language in a following cloze task. This effect was again more pronounced among women than men.

#### 3.6.3 Summary

The available evidence suggests that language use is more susceptible to situational influences (3/3) than attitudes (1/3). It appears possible to encourage gender-inclusive language use through situational cues although with a higher proneness to behavior change in women than men. However, research on this topic remains limited, and additional investigation is required to corroborate these effects.

### 3.7 Interest in gender related topics

An interest in—and a deeper engagement with—gender-related topics, such as the linguistic equality of all genders, could also influence a person’s attitude toward and use of gender-inclusive language. We identified 3 studies on the Swedish gender-neutral pronoun *hen*. All 3 assessed effects on attitudes toward gender-inclusive language, and 2 of those also the influence on language use.

#### 3.7.1 Attitudes

Gustafsson Sendén et al. (2015, 2021) and Renström et al. (2022) showed that greater interest in gender issues was associated with more positive attitudes toward gender-inclusive language. Notably, interest was a stronger predictor of attitudes than political orientation in these studies.

#### 3.7.2 Language use

The studies by Gustafsson Sendén et al. (2015, 2021) also indicate a positive association between a greater interest in gender issues and the more frequent use of *hen*. In fact, in their 2015 study, this interest was found to be the strongest predictor.

#### 3.7.3 Summary

Interest in gender related topics has proven to be a strong predictor of attitudes (3/3) and behavior (2/2) but has exclusively been studied in Swedish-language contexts. Future studies should address this research gap.

### 3.8 Educational level

Gender-inclusive language is often criticized for being harder to understand than masculine generic forms, an assumption that suggests individuals of lower language proficiency and/or a lower level of education could hold more negative attitudes and use gender-inclusive language less frequently than those of higher educational levels. We identified 3 studies in this category, 2 examining effects on attitudes toward gender-inclusive and 2 assessing effects on language use.

#### 3.8.1 Attitudes

Steiger and Irmen (2011) investigated attitudes toward gender-inclusive language in two groups: a group of legal professionals with a university degree and a group of individuals in vocational training without tertiary education. The results showed no differences in the mean attitude scores between the groups. A thematically similar study by Pabst and Kollmayer (2023) asked participants with and without an academic background to evaluate various texts, either using the generic masculine or forms with gender asterisks. Comprehensibility, word and sentence difficulty was rated similarly by both groups and neither depended on the text version nor on attitudes toward gender-inclusive language. This finding contradicts the idea that gender-inclusive language is inherently harder to understand (for converging evidence see also Friedrich and Heise, 2019) and, therefore, less accepted.

#### 3.8.2 Language use

Differences in the frequency of gender-inclusive language use between apprentices and university students have been observed in a German speaking study by Kuhn and Gabriel (2014). Participants completed a language proficiency test followed by a cloze task with nine short passages. Five passages included an initial letter for words referring to gender-fair social roles (e.g., “F” for *Freunde und Freundinnen,* friends_masc_ and friends_fem_ [gender-inclusive] or *Freunde,* friends_masc_ [generic masculine]), two related to professions stereotypically associated with women (e.g., nurse), and two stereotypically associated with men (e.g., firefighter). Additionally, four passages were set in a private and five in a public context. Despite apprentices scoring significantly lower in language proficiency, they used more gender-inclusive forms than university students. However, when both groups were explicitly asked to avoid generic wording, they increased their use of gender-inclusive language, and the differences between groups disappeared. Similarly, the study by Steiger and Irmen (2011) found no evidence supporting a relation between educational level and gender-inclusive language use. In fact, their findings indicated the opposite: compared to participants in vocational training without higher education, those with a university background used the generic masculine more frequently and less frequently opted for neutral or inclusive forms.

#### 3.8.3 Summary

In summary, current results indicate that individuals of lower educational levels and lower language proficiency hold similar attitudes toward gender-inclusive language (2/2) and are as capable of using it as those of higher educational levels (2/2). Further research could substantiate this finding.

### 3.9 Influences with small database

The following factors provide further insight into possible influences on attitudes toward gender-inclusive language and its use. However, the database so far is small. Two studies assessed different aspects of personality on attitudes or language use, each of the other factors was investigated in only one study.

#### 3.9.1 Personality

Parks and Roberton (2005) examined empathy as a potential mediator between age and gender on attitudes toward gender-inclusive language and differentiated between perspective-taking as the cognitive component and empathic concern as the affective component of empathy. The results indicated significant but relatively weak correlations between empathy and attitudes. Perspective-taking was found to be a more influential mediator between age and attitudes among male participants. The authors suggested that increasing age might enhance men’s understanding of those affected by sexist language. Empathic concern, however, had no significant effect. A study by McMinn et al. (1990) investigated the influence of aggressiveness and assertiveness on the frequency of gender-inclusive language use in a written response to a moral dilemma. However, no effect of these traits on language use was found. Research examining the influence of common personality models (e.g., HEXACO model, Ashton and Lee, 2020) on attitudes toward and the use of gender-inclusive language, could be complementarily relevant and insightful.

#### 3.9.2 Motivation for accuracy in language use

Kuhn and Gabriel (2014) hypothesized that the motivation to express oneself accurately in writing should correlate with more frequent use of gender-inclusive language, as this ensures the inclusion of all addressees—unlike the generic masculine. To test this, they measured students’ and apprentices’ language skills and their motivation for accurate language use via a questionnaire and assessed their language use with a cloze test. The results showed no direct influence of accuracy motivation on the use of gender-inclusive language in either group. However, among apprentices, the relationship between proficiency and language use depended on accuracy motivation. Interestingly and against predictions, lower motivation was associated with a stronger positive correlation between proficiency and gender-inclusive language use, whereas higher accuracy motivation strengthened the relationship between low language skills and the use of gender-inclusive language. According to the authors, these findings may indicate that apprentices with high language skills do not consider gender-inclusive language to be precise. The findings of Kuhn and Gabriel (2014) show the need to clarify what constitutes precise language and how gender-inclusive wording can support this aim.

#### 3.9.3 Traditionalism and cisgenderism

Traditionalism and cisgenderism are related to sexism, as they aim to uphold gender stereotypes and, in the case of cisgenderism, discriminate against non-binary individuals. The influence of traditionalism and cisgenderism was examined by Renström et al. (2022) in relation to attitudes toward and the use of the pronoun *hen*. Traditionalism, cisgenderism, and attitudes were measured via self-report, while *hen* usage was assessed using a cloze test. The authors hypothesized that traditionalism would be more negatively associated with the generic than the specific meaning of *hen*, whereas cisgenderism would show a stronger negative correlation with the specific meaning than the generic one. These assumptions were confirmed for participants’ attitudes. However, traditionalism did not predict the use of *hen*, but higher cisgenderism scores were associated with lower use of *hen* in both its generic and specific meanings. Further research is needed on these constructs. The influence of cisgenderism appears particularly relevant in the emerging research on non-binary individuals within the context of gender-inclusive language.

#### 3.9.4 Religious orientation

McMinn et al. (1990) highlighted that religious orientation can play an important role in shaping attitudes toward and the use of gender-inclusive language, since certain belief systems may be linked to traditional gender roles, which in turn can influence linguistic preferences. In their study, McMinn et al. (1990) measured religious beliefs via self-report, while language use was assessed through participants’ responses to a moral dilemma. Participants with less adherence to fundamentalist Christian beliefs used less sexist language in their responses. As before, further research is needed on the influence of religious orientation on attitudes toward and use of gender-inclusive language to better understand this influence.

## 4 Discussion

Reviewing the literature on attitudes toward and use of gender-inclusive language over the last decades shows that sexist beliefs are among the strongest predictors of both attitudes and behavior. Modern sexism in particular emerges as a strong predictor of negative attitudes and lower usage (e.g., Gustafsson Sendén et al., 2015; Sarrasin et al., 2012; Swim et al., 2004). Similar relationships exist for neosexism (Sczesny et al., 2015), and hostile sexism (e.g., Sarrasin et al., 2012). Effects of sexism were observed in different language contexts, English, French, Swedish, and German. No significant associations between benevolent sexism and attitudes or language use were found (Sarrasin et al., 2012; Swim et al., 2004). Increased cognitive load appears to suppress sexism-based differences in language behavior (Cralley and Ruscher, 2005). This underlines that underlying gender stereotypes, reinforced by habitual processes and socialization, are deeply ingrained. When cognitive load is high, the ability to suppress these stereotypes is reduced, leading to an increase in sexist expressions.

The relationship of age to gender-inclusive language depends on the scope and concreteness of what is studied. Younger individuals tend to have more positive attitudes toward the new pronoun *hen* (e.g., Gustafsson Sendén et al., 2015; Renström et al., 2022) but when gender-inclusive language refers to the general avoidance of sexist formulations, older individuals exhibit more positive attitudes than younger ones (e.g., Parks and Roberton, 2008; Rubin and Greene, 1991). No clear conclusion can be drawn regarding which age group uses gender-inclusive language more frequently. A clearer pattern emerges regarding the influence of time/duration of exposure: studies across different linguistic contexts (German, English, Swedish) found an increase in the use of gender-inclusive language over time (e.g., Gustafsson Sendén et al., 2015; Link, 2024; Rubin et al., 1994; Waldendorf, 2024). Regarding attitudes, this positive time effect has only been observed in Swedish, specifically toward *hen*, and only among younger individuals (e.g., Gustafsson Sendén et al., 2021). Moreover, these temporal effects are likely also linked to changes in social and political norms, such as the case of the official inclusion of *hen* in the Swedish dictionary.

Social and political conditions show a relevance for both attitudes toward and the use of gender-inclusive language. Citizens of countries that implemented gender equality laws earlier tend to have more positive attitudes than those of countries where such laws were introduced later (Sarrasin et al., 2012); moreover, gender-inclusive language is more established in countries with greater gender equality (see Hodel et al., 2017). On the level of close social relationships, small positive correlations with empathy suggest that understanding another person’s perspective and situation may promote inclusive language behavior and foster more positive attitudes (e.g., Parks and Roberton, 2002).

Situational influences seem to have predictive power, too. When individuals perceive their interlocutor as supportive of gender-inclusive language, they are more likely to express positive attitudes toward it in that context (e.g., Rubin and Greene, 1991). Similarly, language use varies depending on the audience, with gender-inclusive language being used less frequently in informal than in formal contexts (Rubin et al., 1994). The use of gender-inclusive language can be reinforced through explicit arguments and concrete cues. However, indirect prompting or priming—e.g., through exposure to gender-inclusive language in a text—only influenced linguistic behavior in women (e.g., Koeser et al., 2015; Koeser and Sczesny, 2014). The influence of interest in gender related topics appeared to be relevant for both attitudes toward and use of gender-inclusive language in Swedish (Gustafsson Sendén et al., 2015, 2021; Renström et al., 2022) but should be investigated in other linguistic contexts.

The influence of political orientation on attitudes remains unclear, but its effect on language use is more consistent. Liberal parties and media are more likely to use gender-inclusive language than conservatives (Stecker et al., 2021; Waldendorf, 2024), and right-wing authoritarianism is related to less frequent use of gender-inclusive language (Renström and Klysing, 2024).

The biological sex of a person per se appears to have limited predictive power. Although a number of studies address this variable, the findings are inconsistent. Even though women exhibit more positive attitudes and use gender-inclusive language more frequently than men (e.g., Bruns and Leiting, 2024; Jacobson and Insko, 1985; Parks and Roberton, 2004, 2005), differences are at times small or not statistically significant. Moreover, the predictive power of biological sex and gender diminishes when variables reflecting values or stereotypes, such as sexism or cisgenderism, are taken into account (Gustafsson Sendén et al., 2015; Renström et al., 2022).

Studies show no significant differences in attitudes based on educational level (e.g., Steiger and Irmen, 2011; Pabst and Kollmayer, 2023) and rather point in an unexpected direction when it comes to language use: individuals without an academic background tend to use gender-inclusive language more frequently than those with higher education (Kuhn and Gabriel, 2014; Steiger and Irmen, 2011). These findings are based on German-speaking contexts, highlighting the need for research in other languages.

From the findings, three factors emerge as major influences on attitudes toward and use of gender-inclusive language: a person’s stance on sexism, the context they are immersed in, locally and on a societal level, and passing time. The finding that sexist beliefs strongly influence both attitudes toward and the use of gender-inclusive language underscores how deeply ingrained gender roles remain and how they contribute to the linguistic discrimination of women and non-binary individuals. This emphasizes the relevance of public debate on still existing gender inequality, the relevance of policies and of educational programs in schools to foster a societal culture of gender equality. The reviewed studies suggest that in combination with passing time such measures are likely to improve attitudes toward and the use of gender-inclusive language.

The finding that gender-inclusive language use and attitudes do not depend on educational level may point to one aspect which has not received much attention in the literature so far, namely that language change may be challenging for all who have been socialized in traditional gender-exclusive language (Bruns and Leiting, 2025, report that people with positive attitudes mention how hard it is to change language habits, even though they are motivated to do so), and this seems to particularly lead to resistance toward newly created strategies of gender-inclusive language among older generations. At the same time, language use is extremely complex and highly automatized. Thus, first steps in producing unfamiliar words and phrases require cognitive control which is effortful. Well-founded and well-explained policies as well as practice (and training) in gender-inclusive language appear to be useful measures to pave the way for a more widespread usage of gender-inclusive language. Policies can motivate people to invest the effort because they do not want to behave in a sexist way, and practice (supported through training and/or guidelines) will improve proficiency.

Some factors have too small a database so far to fully grasp their possible influence on attitudes toward gender-inclusive language and its use. Traditionalism and cisgenderism are closely linked to sexism, and cisgenderism may emerge as a particularly influential factor in shaping attitudes toward and a language use that includes non-binary individuals. With regard to personality traits, further research could focus on widely used taxonomies (e.g., the HEXACO model, Ashton and Lee, 2020), where the dimension of openness in particular could be associated with more positive attitudes and more frequent use of gender-inclusive language. Findings on the motivation for accuracy in language use raise the relevant question of what counts as accurate for whom. Since current research increasingly includes non-binary individuals, existing findings on well-documented variables could be further refined in this regard. Finally, a significant gap in the current literature is the lack of studies in non-Western (linguistic) contexts, which needs to be closed to truly grasp what affects gender-inclusive language attitudes and use globally. All of these topics are worth being pursued in future research.

## 5 Conclusion

This review has identified several influential variables affecting attitudes toward and the use of gender-inclusive language. It has also shown some factors that may have been expected to be highly relevant to have no simple effects (biological sex, gender) or to be irrelevant or even go along with more frequent use of gender-inclusive language (low educational level). The existing evidence shows that linguistic behavior is primarily shaped by the habitual reproduction of underlying gender stereotypes which continue to persist in society and manifest in language use. Even individuals who attempt to avoid sexist formulations may still use them—particularly under cognitive load—suggesting the influence of both controlled and habitual processes and the challenge that language change poses. From the existing evidence base regarding gender-inclusive language, we have derived possible strategies to promote its use, and from the research gaps we have defined useful pathways for future studies. Policies and practice matter. Given the infinite possibilities of language to express any subject with precision and independently of context, all existing limitations of gender-inclusive language lie with the users, not with the medium.

## Footnotes

^1^ https://osf.io/p648a/?view_only=a385a4820769497c93a9812d9ea34419

^2^Italy: Ministry of Education and Merit (n.d.), Note prot. no. 1784 “Clarification of the use of the graphic symbol of the asterisk (*) or the schwa (ə) in official communications of educational institutions” (retrieved at https://www.mim.gov.it/-/mim-circolare-alle-scuole-il-ministero-raccomanda-di-non-usare-simboli-nelle-comunicazioni-ufficiali-e-di-attenersi-alle-regole-della-lingua-italiana; 08/14/2025); Germany: Bavarian State Ministry of Justice (n.d.); §22 “Multi-gender spelling using internal word symbols such as gender asterisk, colon, gender gap or media dot are not permitted.“(retrieved at https://www.gesetze-bayern.de/Content/Document/BayAGO-22; 08/14/2025); France: Ministry of National Education, Youth and Sports (2021), NOR: MENB2114203C “Rules for feminization in the administrative acts of the Ministry of National Education, Youth and Sports and teaching practices” (retrieved at https://www.education.gouv.fr/bo/21/Hebdo18/MENB2114203C.htm; 08/14/2025); Translation by authors.

## References

[ref1] Abbondanza M. Galimberti V. Bonomi V. Reverberi C. Durante F. Foppolo F. (2025). Neutralizing gender in role nouns: investigating the effect of ə in written and oral Italian. Front. Commun. 9:1530778. doi: 10.3389/fcomm.2024.1530778

[ref2] Ajzen I. (1991). The theory of planned behavior. Organ. Behav. Hum. Decis. Process. 50, 179–211. doi: 10.1016/0749-5978(91)90020-t

[ref3] Ashton M. C. Lee K. (2020). Objections to the HEXACO model of personality structure—and why those objections fail. Eur. J. Personal. 34, 492–510. doi: 10.1002/per.2242

[ref4003] Bavarian State Ministry of Justice. (n.d.). Allgemeine Geschäftsordnung für die Behörden des Freistaates Bayern (AGO) [General Administrative Regulation for the Authorities of the Free State of Bavaria]. Available at: https://www.gesetze-bayern.de/Content/Document/BayAGO-22 (Accessed: 14 August 2025).

[ref4] Bem S. L. Bem D. J. (1973). Does sex-biased job advertising “aid and abet” sex discrimination? J. Appl. Soc. Psychol. 3, 6–18. doi: 10.1111/j.1559-1816.1973.tb01290.x

[ref5] Bizumic B. Duckitt J. (2018). Investigating right wing authoritarianism with a very short authoritarianism scale. J. Soc. Polit. Psychol. 6, 129–150. doi: 10.5964/jspp.v6i1.835

[ref6] Blaubergs M. S. (1980). An analysis of classic arguments against changing sexist language. Womens Stud. Int. Q 3, 135–147. doi: 10.1016/S0148-0685(80)92071-0

[ref7] Bruns H. Leiting S. (2024). “Using gender-inclusive language in German? It’s a question of attitude” in Public attitudes towards gender-inclusive language: a multilingual perspective. Language and Social Life [LSL] Series: v.31. ed. Pfalzgraf F.. 1st ed (Berlin, Boston: De Gruyter Inc), 97–126. doi: 10.1515/9783111202280-005

[ref8] Bruns H. Leiting S. (2025). Annoying or important? Arguments for and against gender-inclusive language use in German across age and gender. J. Lang. Discrimination 8, 165–191. doi: 10.3138/jld-2024-0103, PMID: 40673054

[ref9] Cacciari C. Corradini P. Padovani R. Carreiras M. (2011). Pronoun resolution in Italian: the role of grammatical gender and context. J. Cogn. Psychol. 23, 416–434. doi: 10.1080/20445911.2011.526599

[ref10] Corbett G. G. (1991). Gender (Cambridge Textbooks in Linguistics). Cambridge: Cambridge University Press. ISBN: 9780521329392.

[ref11] Cralley E. L. Ruscher J. B. (2005). Lady, girl, female, or woman. J. Lang. Soc. Psychol. 24, 300–314. doi: 10.1177/0261927X05278391

[ref12] Dueñas J.-M. Santiago-Larrieu B. Ferre-Rey G. Cosi S. (2020). Ambivalent sexism in adolescence: the relationship between family socialization styles and ambivalent sexism in adolescence. Interpersona Int. J. Pers. Relat. 14, 28–39. doi: 10.5964/ijpr.v14i1.3923

[ref13] Ekehammar B. Akrami N. Araya T. (2000). Development and validation of Swedish classical and modern sexism scales. Scand. J. Psychol. 41, 307–314. doi: 10.1111/1467-9450.00203, PMID: 11131952

[ref14] Formanowicz M. M. Cisłak A. Horvath L. K. Sczesny S. (2015). Capturing socially motivated linguistic change: how the use of gender-fair language affects support for social initiatives in Austria and Poland. Front. Psychol. 6:1617. doi: 10.3389/fpsyg.2015.01617, PMID: 26582996 PMC4628104

[ref15] Friedrich M. C. G. Gajewski S. Hagenberg K. Wenz C. Heise E. (2024). Does the gender asterisk (“Gendersternchen”) as a special form of gender-fair language impair comprehensibility? Discourse Process. 61, 439–461. doi: 10.1080/0163853X.2024.2362027

[ref16] Friedrich M. C. G. Heise E. (2019). Does the use of gender-fair language influence the comprehensibility of texts? Swiss J. Psychol. 78, 51–60. doi: 10.1024/1421-0185/a000223

[ref17] Gabriel U. Gygax P. M. Kuhn E. A. (2018). Neutralising linguistic sexism: promising but cumbersome? Group Process. Intergroup Relat. 21, 844–858. doi: 10.1177/1368430218771742

[ref18] Glick P. Fiske S. T. (1996). The ambivalent sexism inventory: differentiating hostile and benevolent sexism. J. Pers. Soc. Psychol. 70, 491–512. doi: 10.1037/0022-3514.70.3.491

[ref19] Gustafsson Sendén M. Bäck E. A. Lindqvist A. (2015). Introducing a gender-neutral pronoun in a natural gender language: the influence of time on attitudes and behavior. Front. Psychol. 6:893. doi: 10.3389/fpsyg.2015.00893, PMID: 26191016 PMC4486751

[ref20] Gustafsson Sendén M. Renström E. Lindqvist A. (2021). Pronouns beyond the binary: the change of attitudes and use over time. Gender Soc. 35, 588–615. doi: 10.1177/08912432211029226

[ref21] Hausmann R. Tyson L. D. Zahidi S. (Eds.) (2012). The global gender gap 2012: World Economic Forum. Geneva, Switzerland: World Economic Forum. https://www3.weforum.org/docs/WEF_GenderGap_Report_2012.pdf

[ref22] Heider F. (1946). Attitudes and cognitive organization. J. Psychol. 21, 107–112. doi: 10.1080/00223980.1946.9917275, PMID: 21010780

[ref23] Hellinger M. Bußmann H. (Eds.) (2001). Gender across languages (Impact: Vols. 1–3). Amsterdam: John Benjamins Publishing Company.

[ref24] Henley N. M. Dragun D. (1983). Survey of attitudes toward changing sex-biased language [conference paper]. Anaheim, CA: Paper Presented at the American Psychological Association Annual Convention.

[ref25] Hodel L. Formanowicz M. Sczesny S. Valdrová J. von Stockhausen L. (2017). Gender-fair language in job advertisements. J. Cross-Cult. Psychol. 48, 384–401. doi: 10.1177/0022022116688085

[ref26] Horvath L. K. Sczesny S. (2016). Reducing women’s lack of fit with leadership positions? Effects of the wording of job advertisements. Eur. J. Work Organ. Psychol. 25, 316–328. doi: 10.1080/1359432X.2015.1067611

[ref27] Irmen L. Roßberg N. (2004). Gender markedness of language: the impact of grammatical and nonlinguistic information on the mental representation of person information. J. Lang. Soc. Psychol. 23, 272–307. doi: 10.1177/0261927X04266810

[ref28] Jacobson M. B. Insko W. R. (1985). Use of nonsexist pronouns as a function of one's feminist orientation. Sex Roles 13, 1–7. doi: 10.1007/BF00287456

[ref29] Jones K. L. Noorbaloochi S. Jost J. T. Bonneau R. Nagler J. Tucker J. A. (2018). Liberal and conservative values: what we can learn from congressional tweets. Polit. Psychol. 39, 423–443. doi: 10.1111/pops.12415

[ref30] Koeser S. Kuhn E. A. Sczesny S. (2015). Just reading? How gender-fair language triggers readers’ use of gender-fair forms. J. Lang. Soc. Psychol. 34, 343–357. doi: 10.1177/0261927X14561119

[ref31] Koeser S. Sczesny S. (2014). Promoting gender-fair language. J. Lang. Soc. Psychol. 33, 548–560. doi: 10.1177/0261927X14541280

[ref32] Kolek V. (2019). Discourse of non-heteronormative labelling in German-language press: the case of Gendersternchen. Slovenščina 2.0 7, 118–140. doi: 10.4312/slo2.0.2019.2.118-140

[ref33] Kuhn E. A. Gabriel U. (2014). Actual and potential gender-fair language use. J. Lang. Soc. Psychol. 33, 214–225. doi: 10.1177/0261927X13504297

[ref34] Lange E. (2024). Der Einfluss ausgewählter Variablen auf Einstellungen und Verhalten bezüglich gendergerechter Sprache. [bachelor’s thesis]. Essen, Germany: University of Duisburg-Essen.

[ref35] Lee J. F. K. (2007). Acceptability of sexist language among young people in Hong Kong. Sex Roles 56, 285–295. doi: 10.1007/s11199-006-9170-4

[ref36] Link S. (2024). The use of gender-fair language in Austria, Germany, and Switzerland: a contrastive, corpus-based study. Lingua 308:103787. doi: 10.1016/j.lingua.2024.103787

[ref37] Luhtanen R. Crocker J. (1992). A collective self-esteem scale: self-evaluation of one's social identity. Personal. Soc. Psychol. Bull. 18, 302–318. doi: 10.1177/0146167292183006

[ref38] McMinn M. R. Lindsay S. F. Hannum L. E. Troyer P. K. (1990). Does sexist language reflect personal characteristics? Sex Roles 23, 389–396. doi: 10.1007/BF00289227

[ref4001] Ministry of National Education, Youth and Sports. (2021). Règles de féminisation dans les actes administratifs du ministère de l’Éducation nationale, de la Jeunesse et des Sports et les pratiques d’enseignement [Guidelines on feminization in administrative acts of the Ministry of National Education, Youth and Sports and teaching practices]. Bulletin officiel n° 18. Available at: https://www.education.gouv.fr/bo/21/Hebdo18/MENB2114203C.htm (Accessed: 14 August 2025).

[ref4002] Ministry of Education and Merit. (n.d.). Circolare: “Il Ministero raccomanda di non usare simboli nelle comunicazioni ufficiali e di attenersi alle regole della lingua italiana” [Circular: “The Ministry recommends not using symbols in official communications and to adhere to the rules of the Italian language”]. Available at: https://www.mim.gov.it/-/mim-circolare-alle-scuole-il-ministero-raccomanda-di-non-usare-simboli-nelle-comunicazioni-ufficiali-e-di-attenersi-alle-regole-della-lingua-italiana (Accessed: 14 August 2025).

[ref39] Pabst L. M. Kollmayer M. (2023). How to make a difference: the impact of gender-fair language on text comprehensibility amongst adults with and without an academic background. Front. Psychol. 14:1234860. doi: 10.3389/fpsyg.2023.1234860, PMID: 38162962 PMC10755001

[ref40] Parks J. B. Roberton M. A. (1998). Contemporary arguments against nonsexist language: Blaubergs (1980) revisited. Sex Roles 39, 445–461. doi: 10.1023/A:1018827227128

[ref41] Parks J. B. Roberton M. A. (2000). Development and validation of an instrument to measure attitudes toward sexist/nonsexist language. Sex Roles 42, 415–438. doi: 10.1023/A:1007002422225

[ref42] Parks J. B. Roberton M. A. (2001). Erratum: Inventory of attitudes toward sexist/ nonsexist language – general (IASNL-G): A correction in scoring procedures. Sex Roles 44, 253. doi: 10.1023/A:1017376822176

[ref43] Parks J. B. Roberton M. A. (2002). The gender gap in student attitudes toward sexist/nonsexist language: implications for sport management education. J. Sport Manag. 16, 190–208. doi: 10.1123/jsm.16.3.190

[ref44] Parks J. B. Roberton M. A. (2004). Attitudes toward women mediate the gender effect on attitudes toward sexist language. Psychol. Women Q. 28, 233–239. doi: 10.1111/j.1471-6402.2004.00140.x

[ref45] Parks J. B. Roberton M. A. (2005). Explaining age and gender effects on attitudes toward sexist language. J. Lang. Soc. Psychol. 24, 401–411. doi: 10.1177/0261927X05281427

[ref46] Parks J. B. Roberton M. A. (2008). Generation gaps in attitudes toward sexist/nonsexist language. J. Lang. Soc. Psychol. 27, 276–283. doi: 10.1177/0261927x08317956

[ref47] Prentice D. A. (1994). Do language reforms change our way of thinking? J. Lang. Soc. Psychol. 13, 3–19. doi: 10.1177/0261927X94131001

[ref48] Remigio T. R. Talosa A. D. (2021). Student’s general attitude in gender-inclusive language. Int. J. Eval. Res. Educ. 10:864. doi: 10.11591/ijere.v10i3.21573

[ref49] Renström E. A. Klysing A. (2024). Ideological origins of resistance against gender-inclusive language reforms: singular they as a de-gendering or multi-gendering strategy. Polit. Psychol. 1–19. doi: 10.1111/pops.13058

[ref50] Renström E. A. Lindqvist A. Gustafsson Sendén M. (2022). The multiple meanings of the gender-inclusive pronoun hen: predicting attitudes and use. Eur. J. Soc. Psychol. 52, 71–90. doi: 10.1002/ejsp.2816

[ref51] Rothmund J. Christmann U. (2002). Auf der Suche nach einem geschlechtergerechten Sprachgebrauch: Führt die Ersetzung des generischen Maskulinums zu einer Beeinträchtigung von Textqualitäten? Muttersprache 112, 115–136. ISSN: 0027-514X.

[ref52] Rubin D. L. Greene K. L. (1991). Effects of biological and psychological gender, age cohort, and interviewer gender on attitudes toward gender-inclusive/exclusive language. Sex Roles 24, 391–412. doi: 10.1007/BF00289330

[ref53] Rubin D. L. Greene K. L. Schneider D. (1994). Adopting gender-inclusive language reforms. J. Lang. Soc. Psychol. 13, 91–114. doi: 10.1177/0261927X94132001

[ref54] Sarrasin O. Gabriel U. Gygax P. (2012). Sexism and attitudes toward gender-neutral language. Swiss J. Psychol. 71, 113–124. doi: 10.1024/1421-0185/a000078

[ref55] Sczesny S. Formanowicz M. M. Moser F. (2016). Can gender-fair language reduce gender stereotyping and discrimination? Front. Psychol. 7:25. doi: 10.3389/fpsyg.2016.00025, PMID: 26869947 PMC4735429

[ref56] Sczesny S. Moser F. Wood W. (2015). Beyond sexist beliefs: how do people decide to use gender-inclusive language? Pers. Soc. Psychol. Bull. 41, 943–954. doi: 10.1177/0146167215585727, PMID: 26015331

[ref57] Spence J. T. Hahn E. D. (1997). The attitudes toward women scale and attitude change in college students. Psychol. Women Q. 21, 17–34. doi: 10.1111/j.1471-6402.1997.tb00098.x

[ref58] Spence J. T. Helmreich R. L. (1972). The attitudes toward women scale: an objective instrument to measure attitudes toward the rights and roles of women in contemporary society. JSAS Catalog Sel. Doc. Psychol. 2, 66–67.

[ref59] Spence J. T. Helmreich R. L. Stapp J. (1973). A short version of the attitudes toward women scale (AWS). Bull. Psychon. Soc. 2, 219–220. doi: 10.3758/BF03329252

[ref60] Stahlberg D. Braun F. Irmen L. Sczesny S. (2007). “Representation of the sexes in language” In Fiedler K. (Ed.), Social communication. A volume in the series Frontiers of Social Psychology (pp. 163–187). (Series Editors: A. W. Kruglanski and J. P. Forgas). (New York: Psychology Press).

[ref61] Stecker C. Müller J. Blätte A. Leonhardt C. (2021). The evolution of gender-inclusive language. Evid. German Bundestag, 1949–2021 [Preprint]. Open Science Framework. doi: 10.31219/osf.io/fcsmz

[ref62] Steiger V. Irmen L. (2007). Zur Akzeptanz und psychologischen Wirkung generisch maskuliner Personenbezeichnungen und deren Alternativen in juristischen Texten. Psychol. Rundsch. 58, 190–200. doi: 10.1026/0033-3042.58.3.190

[ref63] Steiger V. Irmen L. (2011). Recht verständlich und “gender-fair”: Wie sollen Personen in amtlichen Texten bezeichnet werden? Ein Vergleich verschiedener Rezipientengruppen zur Akzeptanz geschlechtergerechter Rechtssprache. Linguistische Berichte 2011, 44–69. doi: 10.46771/2366077500227_2

[ref64] Stout J. G. Dasgupta N. (2011). When he doesn't mean you: gender-exclusive language as ostracism. Personal. Soc. Psychol. Bull. 37, 757–769. doi: 10.1177/0146167211406434, PMID: 21558556

[ref65] Strafelda E. (2018). Relationships with Women and Attitudes Towards Sexist Language. Retrieved from the University Digital Conservancy. https://hdl.handle.net/11299/197924

[ref66] Svenska Akademien (2015). Svenska Akademiens ordlista över svenska språket [The Swedish Academy’s glossary of the Swedish language] (14th ed.). Stockholm: Norstedt. ISBN: 9789113060125.

[ref67] Swim J. K. Aikin K. J. Hall W. S. Hunter B. A. (1995). Sexism and racism: old-fashioned and modern prejudices. J. Pers. Soc. Psychol. 68, 199–214. doi: 10.1037/0022-3514.68.2.199

[ref68] Swim J. K. Cohen L. L. (1997). Overt, covert, and subtle sexism. Psychol. Women Q. 21, 103–118. doi: 10.1111/j.1471-6402.1997.tb00103.x

[ref69] Swim J. K. Mallett R. Stangor C. (2004). Understanding subtle sexism: detection and use of sexist language. Sex Roles 51, 117–128. doi: 10.1023/B:SERS.0000037757.73192.06

[ref70] Tajfel H. Turner J. C. (1979). “An integrative theory of intergroup conflict” in The social psychology of intergroup relations. eds. Austin W. G. Worchel S. (Monterey, CA: Brooks/Cole), 33–47.

[ref71] Tajfel H. Turner J. C. (1986). “The social identity theory of intergroup behavior” in Psychology of intergroup relations. eds. Austin W. G. Worchel S.. 2nd ed (Chicago, IL: Nelson-Hall Publishers), 7–24.

[ref72] Talosa A. D. (2018). Filipino ESL students androgyny trait, awareness and attitude in gender-fair language. Asian J. Sci. Technol. 9, 8865–8874.

[ref73] Tougas F. Brown R. Beaton A. M. Joly S. (1995). Neosexism: Plus Ça Change, Plus C'est Pareil. Personal. Soc. Psychol. Bull. 21, 842–849. doi: 10.1177/0146167295218007

[ref74] Vergoossen H. P. Renström E. A. Lindqvist A. Gustafsson Sendén M. (2020). Four dimensions of criticism against gender-fair language. Sex Roles 83, 328–337. doi: 10.1007/s11199-019-01108-x

[ref75] Vervecken D. Hannover B. (2015). Yes i can! Effects of gender fair job descriptions on children’s perceptions of job status, job difficulty, and vocational self-efficacy. Soc. Psychol. 46, 76–92. doi: 10.1027/1864-9335/a000229

[ref76] Waldendorf A. (2024). Words of change: the increase of gender-inclusive language in German media. Eur. Sociol. Rev. 40, 357–374. doi: 10.1093/esr/jcad044

[ref77] Zacharski L. Ferstl E. C. (2023). Gendered representations of person referents activated by the nonbinary gender star in German: a word-picture matching task. Discourse Process. 60, 294–319. doi: 10.1080/0163853X.2023.2199531

[ref78] Zajonc R. B. (1968). Attitudinal effects of mere exposure. J. Pers. Soc. Psychol. 9, 1–27. doi: 10.1037/h00258485667435

